# Comment on “Prospective multicenter validation of preoperative plasma ceramides as novel predictive biomarkers for clinically relevant post-hepatectomy liver failure”

**DOI:** 10.1097/JS9.0000000000003036

**Published:** 2025-07-14

**Authors:** Qun Xiang, Wenjing Lin, Jingjing Wang

**Affiliations:** a Department of Infectious Diseases, Hainan Public Health Clinical Center, Haikou City, Hainan, China; b Department of Gastrointestinal Surgery, Hainan Affiliated Hospital of Hainan Medical University, Haikou City, Hainan, China

*Dear Editor,*

We read with great interest the article titled *“Prospective Multicenter Validation of Preoperative Plasma Ceramides as Novel Predictive Biomarkers for Clinically Relevant Post-Hepatectomy Liver Failure*^[1]^*.”* This study presents valuable insights into the potential role of plasma ceramides as biomarkers for predicting post-hepatectomy liver failure, and we commend the authors for their efforts in advancing this important area of research. However, we would like to highlight a few concerns that could impact the robustness and generalizability of the findings. This manuscript was developed following the TITAN Guidelines 2025^[2]^ for the declaration and use of artificial intelligence in scholarly publishing. No AI tools were employed in the conception, design, analysis, interpretation, or writing of this work.

Firstly, although the authors employed LASSO regression for variable selection, the study does not sufficiently address certain clinically relevant factors, such as splenomegaly^[3]^ and the degree of liver cirrhosis^[4]^, which could have significant implications for predicting CR-PHLF (Clinically Relevant Post-Hepatectomy Liver Failure). They are not directly related to circulating ceramides. The exclusion of these variables may undermine the predictive accuracy of the model. Additionally, other known intraoperative factors, such as blood perfusion time and surgery duration^[5]^, were not considered. These factors are crucial, as they could contribute to a more comprehensive understanding of the variables influencing post-hepatectomy liver failure. Ultimately, given the multitude of factors that affect CR-PHLF, isolating a single indicator for its prediction could limit the model’s overall validity. We suggest that these variables could serve as important stratification factors in future models to enhance predictive granularity and clinical applicability.

We respectfully recommend that future studies incorporate a broader range of perioperative variables and consider stratified modeling approaches. Such enhancements would help improve the robustness and generalizability of CR-PHLF prediction tools and support better perioperative clinical decision-making.

## Data Availability

The letter does not have any data.

## Research registration unique identifying number (UIN)

Not registered for the type of the study.
